# Supplementary material for: Are carbon emissions trading and green financial instruments synergistic? -Comprehensive quantitative research based on content analysis
Source: PLoS One. 2024 Mar 7;19(3):e0298601. doi: 10.1371/journal.pone.0298601 (PMC10919601; doi:10.1371/journal.pone.0298601)
Supplement: S1 Data — (ZIP) [file pone.0298601.s001.zip › Data/地级市Z_F工作报告.zip!/╣π╢1⁄2/╜¡├┼╩╨/└┤╫╘╣1⁄2╓┌║┼í╛┬φ┐╦╩2╛▌═°í┐_2019.docx]

各位代表：

我代表江门市人民政府向大会作工作报告，请予审议，并请政协各位委员和列席人员提出意见。

2018年工作回顾

过去一年是全面贯彻落实党的十九大精神的开局之年，是改革开放40周年。市政府坚持以习近平新时代中国特色社会主义思想为指导，全面贯彻党的十九大和十九届二中、三中全会精神，深入贯彻习近平总书记重要讲话精神，在省委、省政府和市委的正确领导下，在市人大及其常委会的监督和市政协的支持下，围绕落实总书记视察广东重要讲话精神、建设“四个走在全国前列”示范城市的目标，积极融入粤港澳大湾区，克服困难，全力稳增长、促改革、调结构、惠民生、防风险，打好防范化解重大风险、精准脱贫、污染防治三大攻坚战。全国文明城市成功复牌；商事制度改革受到国务院的通报激励；成功防御有记录以来登陆我市的最强台风“山竹”，实现人员“零死亡”；全市经济社会高质量发展水平明显提升，基本完成了市十五届人大四次会议确定的各项目标任务。

一、经济发展稳中向好

稳增长举措初见成效。针对经济运行稳中有变、下行压力加大的形势，按照“六个稳”的要求，积极开展“暖企业、促投资”行动，落实市领导挂钩联系重点项目及重点企业制度，举办了促进民营经济发展千人大会，密集出台了我市“民营经济十条”、“实体经济新十条”、“外资十条”、支持企业技术改造、支持企业搬迁改造、推进“小升规”和“个转企”、投资和重点项目奖惩、加强国土资源管理、促进投融资改革、设立共济发展基金等政策组合，增强了企业发展信心，有力推动经济实现稳中有进。预计全市地区生产总值2900亿元，按可比价计算是1978年的100倍，比上年增长7.8%左右；一般公共预算收入243.93亿元，是1978年的120倍，比上年增长9.8%；社会消费品零售总额1408亿元，是1978年的252倍，比上年增长10%；规模以上工业增加值1085亿元，增长9.4%；固定资产投资增长9.5%，其中工业投资增长8.2%，工业技改投资增长8.8%。主要经济指标的增长幅度均排在全省前列。

“招商引资突破年”效果显著。主动走出去，在深圳、北京、香港、澳门、洛杉矶等地举办大型招商推介活动共10多场，签约产业项目数量创近年来最高，成功引进超亿元产业项目145个、总投资649亿元，其中已供地项目56个、投资额270.2亿元；引进超10亿元产业项目21个、总投资341亿元，其中已供地并动工建设项目12个、投资额163亿元，实现各市（区）至少各1个超10亿元产业项目动工建设的目标；在谈超亿元产业项目240个、总投资1322.7亿元，其中超10亿元产业项目32个、总投资680亿元。全市合同利用外资135.03亿元，增长135.86%，实际吸收外资47.45亿元，增长72.33%，增长幅度均创近年新高。粤澳（江门）产业合作示范区、珠西新材料集聚区、深江产业园、台山工业新城拓展区、开平翠山湖科技产业园拓展区等五大万亩园区全部纳入省产业园管理，新增平整土地5080亩。蓬江、鹤山产业集聚地升格为独立的省产业转移园。

产业结构明显优化。一二三产业比重由2017年的7.2：48.1：44.7调整为6.4：49.1：44.5，二产提高了1个百分点。高端装备制造、新一代信息技术、新能源汽车及零部件、大健康、新材料五大新兴产业加快发展，珠江西岸先进装备制造产业带建设指标完成进度排在全省前列。新引进投资超亿元的装备制造业项目61个，投资总额448.84亿元，其中投资超10亿元项目16个，投资总额338.9亿元。中车广东公司股权调整工作取得突破性进展，获得CRH6A三级检修资质，新签订珠三角城际、深圳6号线等订单39.71亿元；台山核电1号机组具备商业运营条件，成为“EPR全球首堆工程”、“中国经验世界共享”的典范。新增省智能制造试点示范项目6个、总数达8个，“省长杯”工业设计总决赛入围和获奖作品数量均排在全省第2位。全国质量强市示范城市创建工作扎实推进，新增工业类、服务业类省名牌产品23个，创历年新高。开平市被授予“中国建筑之乡”称号，恩平市被授予全国首个“中国演艺装备产业基地”称号。

创新发展步伐加快。启动创建国家创新型城市，江门高新区在全国157个国家高新区中综合排名第62位，连续4年实现争先进位，台山市入选首批国家创新型县（市）建设名单。高新技术企业突破1200家，增长70%，增速连续两年排珠三角第1；新增省级工程技术研究中心104家，增量在全省排名第3；科技型小微企业达3126家，增长47.45%；规模以上工业企业研发机构覆盖率达56.53%。新增科技企业孵化器9家、众创空间11家、科技支行10家，分别达到31家、35家、18家。2名企业家获得国家级“科技创新创业人才”荣誉称号。制定实施《关于进一步集聚新时代人才建设人才强市的意见》，“人才管家”项目获中组部肯定，新引进博士后40人，在站博士后人数比上年翻了一番，新设立2家国家级博士后科研工作站，实施“百名博（硕）士引育工程”，各类人才资源总量突破100万。江门人才岛首期项目顺利开工，完成年度投资25亿元。高技能人才连续8年每年增加万人以上，总数达13.68万人。

服务业发展势头良好。金融服务实体经济能力进一步增强，江门农商银行和鹤山农商银行挂牌营业，江门农商银行成为全省第一家由城区农商银行合并组建的地市级农商银行，总资产达1000亿元，排全省农合机构第6，全市农合机构改革总体进度排在全省前列。编制了《江门市会展业发展规划（2018—2025年）》，大力推进珠西物流枢纽中心建设，鹤山—盐田港海铁联运试运行。积极对接港珠澳大桥开通，大力推进江澳两地世界文化遗产旅游项目等合作活动。台山市全域旅游工作经验在全省推广，广东省首个国家农业公园——台山中国农业公园一期项目建成开园，开平市赤坎古镇项目动工建设，仓东文化遗产保育与发展中心被评定为全国第二批港澳青少年内地游学基地。新增2家国家4A级旅游景区，全市4A级景区达10家。全市全年接待游客6493.1万人次，旅游总收入达587.5亿元，分别增长13.43%和19.28%。

二、改革开放持续深化

区域发展开拓新局。进一步理清区域协调发展思路，积极推动都市核心区、大广海湾区、生态发展区“三区并进”。完成《江门市城市总体规划（2017—2035年）》阶段性成果，编制了《江门市城镇开发边界划定》《市区足球场地设施建设规划》《市区自行车系统专项规划》等10项专项规划。控制性详细规划覆盖率进一步提高。完成县域乡村建设规划编制，基本实现村庄规划全覆盖。

供给侧结构性改革扎实有效。深入推进财政预算体制改革，“去降补”工作加快推进，政府债务等重点领域风险防控工作成效显著，PPP工作在全省领先。国有资产实现保值增值，城投、交建两大平台公司有序运营。进一步降低企业经营成本，为企业减负超过100亿元。市重点项目完成投资783.6亿元，为年度投资计划的126.8%。交通大会战投资170亿元，增长5%。江湛铁路建成通车，江门融入了全国高铁网，可以直达北京、上海；珠西综合交通枢纽江门站站房和配套工程全面动工，南沙港铁路加快建设，完成江门北站货场一期扩建。佛开高速南段昆东至共和立交段完成扩建，高恩高速、中开高速、开春高速、开阳高速扩建等项目加快推进，对接港珠澳大桥的黄茅海通道规划建设工作取得重大进展。江门大道茶坑隧道至三江段、银鹭大桥建成通车，江门大道南东线全面开工建设，改造国、省道97.07公里。完成《江门市综合交通一体化规划(2018—2035年)》编制。公交车纯电动化率超过50%，实现村村通班车。

“放管服”改革走在前列。持续深化清单制、承诺制、委托制改革，投资项目承诺制改革经验在全省推广，前期工作平均提速1—2个月。“证照分离”改革全域推进134项涉企后置审批分类改革，业务量占全省5个试点区域业务总量超八成，“多证合一”改革持续深化，商事登记实现“同城通办、境外可办、网上能办”，开办企业从13.9个工作日压缩至5个工作日以内，开办企业便利度排全省第5。成为全国跨部门“双随机一公开”联合监管试点市、全省首批实施社会信用联合奖惩试点。在全省率先建成网上中介服务超市，率先完成不动产交易、缴税、登记集成服务改革，“数字政府”综合改革列入全省试点。全国第四次经济普查前期工作全面开展。营商环境的优化激发了市场主体活力，全市新发展市场主体19.29万户，增长200.16%，主体总量达47.5万，在全省排名上升至第6位。

开放水平不断提升。成功在美国举办第六届世界江门青年大会，促成涉侨涉外经贸合作项目25个，总金额145亿元，新设立13个境外国际经贸代表机构、累计达19个。江港澳合作进一步深化，分别在香港、澳门举办了“携手共建粤港澳大湾区推介会”系列活动，与澳门特区政府就建设澳门与江门跨境合作实验区达成初步共识；在全国率先实现外资备案登记在港澳离岸远程办理。举办中国（江门）首届侨乡文博会，“少年中国说”系列活动成为粤港澳青少年交流合作重点项目。积极应对中美贸易摩擦，推动口岸提效降费，整体通关时间同比压缩三分之一，完成外贸进出口总额1473亿元，增长6.4%。开平市水口镇、鹤山市址山镇成为国家水暖卫浴外贸转型升级基地。

乡村振兴取得实质性进展。推进农业供给侧结构性改革，广东江门国家农业科技园区成为国家级平台，新增2个省级和10个市级现代农业园，创建档次和数量居全省前列。国家级重点农业龙头企业实现“零的突破”。台山市斗山镇、开平市金鸡镇创建国家农业产业强镇。新增省名牌产品（农业类）17个、累计达62个。“粤菜师傅”工程取得阶段性成效。耕地保护考核连续两年获全省一等奖，高标准基本农田建设连续五年获省政府表彰奖励。大力开展“大棚房”问题和违法违规侵占山水林田湖资源的整治工作。全域推进1108个行政村（社区）人居环境整治，基本完成“三清三拆三整治”工作任务，启动建设15个乡村振兴市级试点示范镇（街）和30个美丽宜居村。“厕所革命”工作扎实推进，新建、改造农村公厕2424座。全面完成农村土地确权登记颁证工作，基本完成新会区国家农村集体产权制度改革试点和鹤山市国家级扶持村级集体经济发展试点任务。

三、民生事业水平提升

民生投入持续加大。全市各级财政投入民生259.05亿元，增长13.7%。省、市十件民生实事顺利完成，“六项民生改革”深入推进，人民群众获得感、幸福感、安全感明显增强。预计居民人均可支配收入2.9万元，增长8%以上。全市城镇新增就业4.88万人，城镇失业人员再就业3.34万人。扶贫工作连续两年在全省扶贫考核中被评为最好等次，基本完成建档立卡贫困户脱贫任务。扶贫线与低保线“两线合一”机制不断完善，城乡最低生活保障标准提高到800元/人/月，实现了全市城乡低保标准一体化。全市新建长者食堂11间，实现每个市（区）至少有一间。超额完成省下达的住房保障任务。

环境治理力度空前。全面落实中央环保督察及“回头看”、省环保督察整改工作，261件“回头看”交办案件全部办结。出台《江门市打好污染防治攻坚战三年行动计划（2018—2020年）》《江门市扬尘污染防治管理办法》《江门市打赢蓝天保卫战2018年工作方案》《江门市全面推行湖长制实施方案》等措施，扎实开展“铁腕治污百日行动”、“清废除患2018”专项行动、“尖兵1号”——潭江流域沿岸工业企业环境执法专项行动。主城区重点行业企业淘汰搬迁工作扎实推进，完成关停117家，搬迁或正在搬迁12家，狠抓燃用高污染燃料设施和“散乱污”企业淘汰工作，市区空气质量在全省排名同比上升3位，PM2.5指标明显改善。压实河长、湖长责任，积极推进西江、潭江流域综合治理，流域内重点企业污染物排放总量减排三分之一以上，在全省率先开展河道“五清”专项行动，全市生猪存栏量压减至100万头，完成41座城镇污水处理厂和1358个农村污水处理设施站点的建设，市区6条黑臭水体水质明显改善，全面推行河长制工作得到国家部委的肯定，在全国专题培训班作经验介绍。深入开展固体废物约谈整改工作，严厉查处111宗涉固体废物违法犯罪案件，一批固体废物焚烧、填埋和利用设施加快建设。在全省率先完成地市级饮用水水源地环境问题整治任务。《市区山体保护规划修编》将市区近半数山体纳入保护规划。创建国家森林城市成果进一步巩固，生态公益林扩面17万亩，完成7个市属、4个县属国有林场改革并通过省级验收，5个镇（街）被认定为“广东省森林小镇”。新增省级绿色矿山7个、省级森林公园2个、省级湿地公园1个。

社会事业全面进步。17所义务教育“三二一”工程学校全部建成，提供优质学位2.7万个。在全省首创编写和推广使用《中国梦·我的梦——习近平新时代中国特色社会主义思想学习读本》丛书。新成立2个市直学前教育集团和蓬江区6个义务教育集团，新办5所优质民办学校。国家基本公共卫生服务考核和城市公立医院综合改革效果考评均排全省第5，全省计生目标管理责任制考评“优秀”，16家紧密型医联体试点分院建设基本完成，大力推进基层医疗服务能力提升工程，家庭医生签约服务提质增效。完成国家农产品质量安全市创建验收，顺利通过省食品安全示范城市中期评估。建成全市首家人力资源服务产业园。企业职工基本养老保险省级统筹、机关事业单位养老保险、医保城乡一体化、医保支付方式等改革工作平稳有序，启动实施按病种分值付费。江门职业技术学院成为省示范性高等职业院校，筹设广东江门幼儿师范高等专科学校获省正式批复同意。全市行政村（社区）综合性文化服务中心实现全覆盖。36个镇（街）开展社会治理公益创投。江门体育中心投入运营，举办了首届江门市民运动大会，承办了一批国际、国内大型赛事。重点优抚对象抚恤补助标准平均提高12.5%，江门市复退军人服务中心新址正式启用。扫黑除恶专项斗争深入推进，全市刑事治安警情、刑事立案数明显下降。各类生产安全事故起数、死亡人数、受伤人数均实现两位数下降，全年未发生较大以上生产安全事故。

城市面貌焕然一新。投入224.18亿元开展城市品质提升“八大行动”（含新区建设），完成了一批城市基础设施建设，开展道路“白改黑”、旧建筑外立面改造、公园“拆围透绿”、社区综合整治，以及蓬江城市中央活动区、江海城央绿廊、新会中心园林区和东湖公园亮化工程。市区新增机动车停车泊位超过4000个。新建、改造城市公厕104座。“三旧”改造新增实施5156亩、完成2967亩，超额完成省下达任务。开展百日攻坚拆违行动，共拆除违建103万平方米。

此外，与广西壮族自治区崇左市的扶贫协作、与四川省甘孜州的对口支援、与黑龙江省七台河市的对口合作等工作扎实推进。外事、港澳、对台、民族、宗教、双拥、人防、打私、统计、粮食、妇女儿童、新闻、出版、档案、地方志、气象、红十字会、应急等工作进一步加强。

四、政府建设不断加强

扎实抓好政府系统党的建设，制定实施《中共江门市人民政府党组关于中央第十二巡视组反馈意见的整改落实方案》《中共江门市人民政府党组工作规则（试行）》，推动党组工作制度化、规范化。基本完成政府系统机构改革任务。落实中央八项规定精神，市本级财政拨款行政事业单位三公经费支出下降6.8%。加强政府系统党风廉政建设，严格落实“一岗双责”，推进审计监督全覆盖，认真落实市委巡察整改工作，坚决查处违纪违法案件。推进“马上就办”，进一步改进政府部门的机关作风。

自觉接受人大和政协的监督，办理人大代表建议和政协提案满意率均达100%。市政府常务会议每季度学法一次，依法行政考评取得近年最好成绩。全市100%的镇（街）、100%的民主法治村（社区）达到省级法治创建标准。政府决策的民主化、科学化、法治化水平进一步提高。

各位代表，过去一年全市经济社会发展所取得的显著成绩，根本在于有习近平新时代中国特色社会主义思想的科学指引，关键是在市委的领导下，在市人大、市政协的大力支持和港澳台同胞、海外侨胞的关心帮助下，全市人民、海内外投资者建设者以及驻江门军警部队共同努力的结果！在此，向参与、支持和关心江门发展的各界人士，表示崇高的敬意和衷心的感谢！

我们也清醒看到，江门经济社会发展中仍然存在许多不平衡不协调不充分的矛盾和问题：一是预计完成生产总值、固定资产投资的增幅，略低于市十五届人大四次会议确定的目标。虽然有经济下行压力大、宏观政策调控等外部原因，但主要还是有的市（区）、部门抓高质量发展的责任不到位、决心和恒心不足、办法和力度不够，个别市（区）的经济指标完成情况与目标相比存在较大的缺口。二是城乡区域发展不平衡不协调。东部不强、西部更弱，东部对西部、中心城区对周边地区未能发挥辐射带动作用。城乡二元结构问题突出，农业产业化水平不高，农村建设投入欠账较多。城市建设和管理水平不高，城镇老旧小区改造力度不大。三是民生领域还有不少短板和欠帐。环境污染治理任重道远，潭江水环境污染状况还没有得到根本好转，牛湾国考断面水质稳定达标任务艰巨，污水处理设施建设滞后，空气质量在全省排名仍然靠后，固体废物处置设施建设短板明显。随着“二孩”政策全面实施以及人口老龄化，教育、医疗卫生、养老等公共服务供给不足，公共文化事业建设有待加强。安全生产风险隐患不容忽视，水利工程整体防灾减灾能力不高。四是一些干部干事创业精气神不足、能力不强、本领不够，庸政懒政怠政现象仍然存在，党风廉政建设任务依然艰巨。对这些问题，我们要勇于面对，采取坚决有力措施加以解决。

2019年工作安排

2019年是新中国成立70周年，是贯彻落实习近平总书记视察广东重要讲话精神、决胜全面建成小康社会、实施“十三五”规划承上启下的关键一年。今年市政府工作的总体要求是：高举习近平新时代中国特色社会主义思想伟大旗帜，全面贯彻党的十九大、十九届二中、三中全会以及中央经济工作会议精神，深入贯彻落实习近平总书记对广东重要讲话和对广东工作一系列重要指示精神，认真贯彻落实省委十二届六次全会、市委十三届九次全会精神，认真落实省委“1+1+9”工作部署和市委“1+1+5”工作举措，坚持稳中求进工作总基调，以供给侧结构性改革为主线，在“巩固、增强、提升、畅通”上下功夫，积极融入粤港澳大湾区规划建设，深化改革开放，推动高质量发展，提高发展平衡性和协调性，加强党的领导和党的建设，继续打好防范化解重大风险、精准脱贫、污染防治三大攻坚战，统筹推进稳增长、促改革、调结构、惠民生、防风险工作，进一步稳就业、稳金融、稳外贸、稳外资、稳投资、稳预期，加快构建“三区并进”区域发展新布局，打造珠江西岸新增长极和沿海经济带上的江海门户，建设“四个走在全国前列”示范城市，以优异的成绩庆祝中华人民共和国成立70周年。

今年全市经济社会发展主要预期目标是：地区生产总值增长7%—7.5%，规模以上工业增加值增长8%，固定资产投资增长9%，社会消费品零售总额增长8.5%，外贸进出口总额增长3%；地方一般公共预算收入增长6.5%；居民消费价格指数涨幅控制在3%以内；居民人均可支配收入增长与经济增长基本同步；城镇新增就业4.5万人，城镇登记失业率控制在3.2%以内。为此，要着力抓好以下工作：

一、以推动大湾区建设为引领深化改革开放

加快构建互联互通现代综合交通网。年度安排交通大会战项目94项，投资175亿元。积极融入大湾区轨道交通网络。全面加快珠西综合交通枢纽江门站站房和配套工程建设，力争2020年春节前基本建成并投入使用，加快南沙港铁路建设，积极争取深茂铁路深圳至江门段动工，推进珠江肇高铁、南深高铁、广佛江珠城际等项目前期工作，加快城市轨道交通示范线建设规划编制。加快高快速道路建设。中开高速完成年度投资25亿元，高恩高速、佛开高速南段扩建建成通车，加快开春高速建设、开阳高速扩建，尽早启动银洲湖高速建设和江鹤高速扩建，加快推进黄茅海通道、南海至新会高速、广台高速开平至台山段等项目前期工作。加快江门大道南、台开快速路、会港大道、南山路、五邑路扩建等快速路建设，启动国道G240线新会段和台山大江至那金段改扩建工程。积极参与粤港澳大湾区港口群建设。争取高新区公共码头一期完成主体工程建设并投入使用。加强江门北站与深圳盐田港、中铁建设等企业的合作，加快“无水港”（启动区）建设并实现海铁联运，打造珠西物流枢纽中心。积极引入广州港等战略合作伙伴，整合和提升港口规划建设水平。推进广海湾深水港、崖门出海航道二期工程等前期工作。规划布局江门主城区和台开至珠三角新干线机场的快速路，推进恩平、台山通用机场的前期工作。

加强与大湾区城市群的合作对接。主动服务港澳发展，全方位深化江港澳在科技、教育、金融、旅游、高端服务业等优势和特色领域的合作，积极推进澳门与江门跨境合作实验区，深化银湖湾滨海新城规划。完善便利港澳居民在江门居住、学习、创业、就业、商旅等配套政策，积极引进港澳优质公共服务。优化大广海湾产业规划，加快广海湾临港产业区建设，谋划广海湾LNG接收站等重大能源项目。积极对接延伸“广深港澳”科技创新走廊，扎实推进珠江西岸先进装备制造产业带建设，谋划打造江珠高端产业集聚发展区，建设大湾区科技创新成果转化基地。积极融入粤港澳大湾区文化圈，打造具有国际影响力的华侨华人文化交流重大平台。

积极扩大对外开放。充分发挥江门的华侨资源优势，加强与“一带一路”沿线国家和地区的经贸合作。打造好国家级平台载体“侨梦苑”，吸引华侨新生代回来创业创新。打造好世界江门青年大会品牌，不断发展壮大五邑青年社团联盟，积极发挥我市驻外经贸代表机构的作用，助推江门企业“引进来”、“走出去”。加强外贸品牌和质量建设，举办2019年“一带一路”进口商品展销会。复制推广自贸区经验，推进口岸提效降费，加快建设大广海湾保税物流中心（B型）。

深化供给侧结构性改革。巩固“去降补”成果，增强微观主体活力，提升产业链水平，畅通国民经济循环。着力补齐交通、电力、水利、通信等领域的短板，加快推进江新联围达标加固和潭江河流治理工程，全面启动西江、潭江重点支流综合整治。统筹推进主城区重污染、高能耗企业关停或搬迁改造。发展壮大国资国企，做大做强城投、交建两大平台公司，积极推进市汽运集团、市碧源公司等国有企业上市。壮大地方金融机构，推进台山、开平农商银行开业。健全政府性债务风险预警机制，防范化解金融风险，创新基础设施和公共服务领域政府投资项目资金的筹措方式。加快房地产综合管理信息系统建设。大力盘活存量低效用地，加快“三旧”改造特别是镇村级工业园升级改造、城中村改造，加大开展城乡增减挂钩和拆旧复垦力度，推进“亩产论英雄”的用地管理体制。划定工业控制线，加快推进垦造水田工作，加大对实体经济、民营经济发展用地保障力度。继续抓好第四次全国经济普查和第三次全国国土调查工作。

系统推进营商环境综合改革。坚持“人人皆为环境”理念，推动各职能部门联动破解企业发展和群众办事的难点痛点堵点，形成打造良好营商环境的合力。深化“证照分离”改革，开展企业开办全程网上办试点工作，推行商事登记智能审批，确保开办时间压缩至3个工作日内，力争开办企业便利度进入全省最前列；完善市场主体退出机制。深化投资项目承诺制改革，加快基准承诺标准的编制进度，争取走在全国前列，全年承诺制管理的投资项目突破100个，力争投资5亿元以上工业投资项目全部实行承诺制。上半年基本建成全市统一的工程建设项目审批和管理体系，加快推进联合审图等配套措施。落实企业投资项目分类管理和落地便利化改革，实现开工前政府审批时间民用建筑项目不超过41个工作日、工业项目不超过45个工作日。深入推进网上中介服务超市运营，加强招标、政府采购市场管理。推进“双随机一公开”监管抽查常态化、制度化。加快构建全市信用联合奖惩一张网。

大力建设人才强市。积极对接大湾区人才高地建设规划，进一步完善人才新政体系。高质量建设江门人才岛，完成年度投资50亿元，大力推进环岛景观带升级改造、国际教育培训中心、人才公寓等项目建设。加快全国博士后创新（江门）示范中心建设。深入实施“百名博（硕）士引育工程”、博士后挂职工程，推进“五邑人才工程”、“侨都英才计划”。积极延伸和扩大国家大科学装置——江门中微子项目在新材料研发、高端人才等的辐射作用。全力支持五邑大学创建省高水平理工科大学、江门职业技术学院创建省高水平高职院校，全力推动江门市技师学院申报高等职业教育，加快现代职业教育综合改革示范项目建设，推动“校企合作”定向培养实用人才。

二、加快构建现代化经济体系

不断增强创新动力。统筹推进创建国家创新型城市与建设珠三角（江门）国家自主创新示范区的工作，全面落实创新驱动发展“八大举措”。推动江门高新区提质升级，完善“一区多园”管理模式，支持台山工业新城、开平翠山湖科技产业园、鹤山工业城创建省级高新区。强化企业创新主体地位，高新技术企业总数突破1300家，规上工业企业研发机构覆盖率超过60%，支持高等院校、科研机构、行业龙头企业等组建国家级、省级技术创新中心或重点实验室。推进政产学研合作，加快融入珠三角国家科技创新成果转移转化示范区，强化知识产权创造、保护和运用。

打造高质量产业平台。加快五大万亩园区建设，新增“三通一平”土地4000亩，提升园区基础设施建设水平，完善生产生活配套，打造大湾区产业对接合作重大平台。持续推进现有核心园区提质增效，加强园区闲置和低效用地的清理，推进产城融合。进一步理顺江门产业转移园管理机制，推进蓬江、江海、开平、鹤山、恩平产业园申请扩大区域。积极推进各类开发区和特色小镇建设。

培育壮大新兴产业和先进制造业。聚焦五大新兴产业，成立重大项目招商专责小组和产业顾问团队，积极破解重大项目招商融资难题。支持各市（区）在重点招商区域设立驻点招商机构和代理招商工作平台，开展精准招商、以商引商、以会招商，深入推进“招商引资突破年”工作，争取各市（区）年内均引进投资额20亿元以上的项目并动工建设，力争引进超50亿元、超百亿元的特大项目。充分发挥市招商引资和工业园区开发建设两个联席会议制度的作用，落实招商引资项目行政审批代办制，全力推动一大批项目开工建设。抓好龙头骨干企业培育，加快发展壮大中车广东公司，推动新会区中集智能物流装备、鹤山市蔚海IDC大数据基地等项目建设，支持优美科新能源汽车锂电池材料等项目建成投产。

推动传统产业高质量发展。提升机电、食品、电子信息、纺织服装等传统产业发展水平，落实省、市技改奖补政策，支持引导企业开展技术改造和增资扩产，大力实施“机器人应用”工程，搭建产业技术公共服务平台。支持智能云科等工业互联网平台建设，探索新一代人工智能技术与制造业深度融合。积极承接大湾区生产性服务业溢出，推动先进制造业和现代服务业深度融合，加快发展工业设计、电子商务、会展业、现代物流、科技和信息服务，积极支持商业综合体发展。深入实施商标品牌战略，加快珠西综合质量检测服务平台建设，扎实推进全国质量强市示范城市创建。

激发民营经济发展活力。继续深入开展“暖企业、促投资”行动，确保各项扶持措施落实到位，充分发挥各级工商联、商会、行业协会桥梁纽带作用，建立健全企业参与政策制定机制，弘扬企业家精神，增强企业家信心。切实减轻民营企业负担，鼓励金融机构加大对民营企业信贷支持力度，进一步简化中小微企业“政银保”融资程序。支持民营企业在境内外上市、融资。做好全国小微企业信用体系建设试验区工作，深化小微企业创业创新工作，大力推动“个转企”、“小升规”。

加快全域旅游发展。编制实施《江门市国际特色旅游目的地全域旅游发展规划（2018—2030年）》，用好侨乡资源，积极对接港珠澳大桥开通的机遇，深化江港澳合作，探索全域旅游的江门新路径，台山市和开平市加快创建国家全域旅游示范区。推动各市（区）积极创建A级旅游景区，抓好碉楼文化旅游区创建国家5A级旅游景区工作，加快开平市赤坎古镇、鹤山市华侨城古劳水乡等龙头旅游项目建设，高水平规划川岛开发建设，提升温泉旅游项目品质，推进省滨海旅游公路江门段前期工作。推动每个市（区）建设1—2个红色旅游景区（景点）。统筹文化、体育、旅游事业深度融合发展。

三、推进区域协调发展

全面推动“三区并进”。对接落实《粤港澳大湾区发展规划纲要》和省“一核一带一区”发展新格局，制定我市专项实施方案。优化全市空间发展布局。科学划定城镇开发边界，细化落实都市核心区、大广海湾区、生态发展区的空间布局、功能定位和发展目标。都市核心区要突出深度一体化，加强对蓬江、江海、新会、鹤山交界区域的产业布局和基础设施规划，加快台开两个中心城区的交通通道建设。大广海湾区要整合资源，串珠成链，江海联动，大力发展海洋经济、临港经济，争取承接一批国家重大项目。生态发展区要在绿色发展上多做文章，因地制宜发展绿色低碳新型工业、现代农业、旅游业等，推动恩平工业园等现有园区加快产业结构调整升级。根据不同的功能区定位，探索实施相应的产业、土地、财政等政策。推动市本级与各市（区）财政管理体制改革。进一步理顺和调整财政体制，解决财政体制碎片化问题，发挥财政体制机制激励作用，形成共促发展、共享成果的财政体制机制。制定《江门市潭江流域生态保护补偿办法》，采用下游补偿上游的模式，建立与区域发展定位相适应的财政转移支付政策，促进区域城乡协调发展、生态文明建设和推进基本公共服务均等化。

加快实施乡村振兴。多措并举促进乡村产业兴旺。深入推进农业供给侧结构性改革，打造18个产值超10亿元的产业联合体，发展农业综合体、乡村旅游等农业新兴产业。积极培育新型农业经营主体，新增市级以上重点农业龙头企业10家、农民专业合作社市级示范社10家，加强与农业科研机构的合作。加快建设广东江门国家农业科技园区、新会陈皮国家现代农业产业园、广东省农产品加工示范区、台山市鳗鱼省级现代农业产业园、开平市家禽省级现代农业产业园和大湾区农产品交易流通中心等现代农业发展平台。新增建成高标准基本农田32.95万亩，落实粮食安全责任制。持续推进全域农村人居环境整治。深入实施“百村示范、千村创建、万村整治”工程，启动干净整洁农村人居环境整治行动，创建100个农村人居环境整治示范村，加快推进15个乡村振兴市级试点示范镇（街）、50个示范村的建设。建设14条美丽乡村精品线路，推进生态宜居美丽乡村示范创建。推进“厕所革命”，全面实现每个自然村按实际需求基本建成1个标准化公厕。高标准、高质量推进“四好农村路”建设，蓬江区争创省级示范县。继续抓好“大棚房”问题专项清理整治。深化农村综合改革。全面推进省级乡村振兴综合改革试点工作和农村集体产权制度改革，深化农村土地确权颁证成果应用。多措并举落实乡村振兴资金投入机制，实施《江门市农村集体资金资产资源管理办法》，堵塞“村账镇管”的制度漏洞。

深化和提升城市品质。深入推进全国文明城市创建工作。实施深化全国文明城市创建工作三年总体方案，推进文明城市创建常态长效，推动台山市、鹤山市创建全国文明城市，开平市创建全国文明城市提名城市，恩平市创建省文明城市，打造珠三角文明城市群示范区。以“绣花功夫”完善城市基础设施和公共服务。加快蓬江城市中央活动区、江海城央绿廊、新会中心园林区三个片区的建设，推动城市“美化、绿化、亮化、净化”持续升级，提升城市出入口景观。加快老旧城区更新改造，活化利用历史街区和建筑，抓好江礼桥以北“三旧”改造项目。研究启动大长江集团旧厂区升级改造和新厂区规划建设。推进老旧城区住宅加装电梯工作。新建和升级改造城市公厕100座。推进市区环卫作业一体化，加快市区餐厨垃圾项目建设。建立长效机制确保违法建设“零增长”。加快提升公共交通水平。完善城市快速路网、步行和自行车等慢行系统建设，打通断头路、瓶颈路，市区新增机动车停车泊位2500个。完善公交一体化发展，公交车纯电动化率达到80%。

四、保障和改善民生

优先发展教育事业。确保义务教育“三二一”工程学校安全投入使用。进一步完善集团化办学，增加优质学位供给。推动学前教育优质普惠发展，落实幼儿园2018—2022年建设专项规划，制定公办幼儿园生均拨款标准和普惠性民办幼儿园补助标准，应对学前教育入园高峰的到来。推动义务教育均衡一体化发展，小学适龄儿童入学率达到99.99%，逐步解决“大班额”问题，落实教师“县管校聘”管理改革。落实高水平高质量普及高中阶段教育任务，高中阶段毛入学率达到98%以上。建设高素质教师队伍，推进教育信息化建设。江门幼儿师范高等专科学校新校区完成一期工程建设并投入使用。加快推进民办教育规范管理，促进特色优质发展。实施第二期特殊教育提升计划。推动开放大学系统转型升级，支持办好老年教育。

积极建设“健康江门”。深化医药卫生体制改革，推进紧密型医联体建设，加快强基项目建设进度。推进公立医院综合改革，建立现代医院管理制度，全面取消药品、医用耗材加成，完善公立医院补偿机制。打造珠西医疗中心，争取建设全省高水平医院，建设江门市紧急医学救援指挥中心。促进社会办医，打造江门国际健康城。加快区域卫生健康信息平台建设，实施“互联网+医疗健康”行动计划。建立健全基层医疗卫生机构财政补偿机制，落实基层卫生人才队伍建设三年行动计划。推进国家基本公共卫生服务，做实家庭医生签约服务。稳步推进社会心理服务体系建设国家试点工作。深入开展“双安双创”，深化国家农产品质量安全市创建工作，全域创建省食品安全示范城市。

提高就业和社会保障水平。鼓励多渠道多形式就业，全市城镇失业人员再就业3万人，推进人力资源服务产业园、创业孵化基地等就业创业载体建设运营。支持本地大学生、农民工回城返乡就业创业，积极实施“粤菜师傅”工程。全面落实企业职工基本养老保险省级统筹，提升基本养老保险待遇，继续推进医保城乡一体化改革，推进工伤保险省级统筹。支持养老产业加快发展，实施养老机构服务质量提升工程和居家社区养老服务“大助餐”工程，深化敬老院公建民营社会化改革。加强农村留守儿童关爱保护和困境儿童保障，完善未成年人保护工作体系。加强残疾儿童康复救助工作，加大扶持残疾人就业创业力度。完善复退军人服务体系的建设，积极筹备新一轮全国双拥模范城创建工作。

推动生态环境质量改善。加快创建国家生态文明建设示范市。按期完成中央环保督察及“回头看”反馈意见、省环保督察整改。打赢蓝天保卫战。深入推进高污染高排放企业淘汰搬迁和“散乱污”企业综合整治，抓好挥发性有机物整治和减排，加强对工地、道路扬尘及露天焚烧的监管，全面落实不利气象条件下大气污染防控联动工作机制，实现空气质量持续好转。打赢碧水攻坚战。严格落实河长制、湖长制，以确保潭江牛湾断面2020年达到II类水质为目标，开展潭江水质攻坚战，建立镇级环保巡查机制，加强控源截污、生态修复和环保监管执法；推动西江水质持续向好。完成县级水源地保护整治工作，保障饮用水安全。市区现有的6条黑臭水体全面消除黑臭现象，新发现的黑臭水体全面进入整治阶段。加快城镇污水处理厂和配套管网建设，实现全市镇级污水处理设施全覆盖，扩大农村污水处理设施覆盖面，启动潭江重点支流沿线重点村庄生活污水整治。打赢净土防御战。做好重点行业企业用地详查，加快全市固体废物特别是危险废物处理设施建设，加强固废管理。保护海洋环境。深入推进国家海洋督察反馈问题整改落实，严格管控围填海，启动海岸线生态修复和美丽海湾项目建设。持续深化森林城市建设。推进新一轮绿化大行动，完成生态公益林扩面17万亩，扎实推进桉树人工林改造和饮用水源水库第一重山商品林改造工程。加快推进新会区小鸟天堂、开平市孔雀湖、台山市镇海湾红树林等三个国家级湿地公园以及各市（区）湿地公园、森林公园、镇（街）公园的建设，完善村居公园管理，统筹做好自然保护地管理工作。

打赢精准脱贫攻坚战。全面推进扶贫线与低保线“两线合一”，建立长效帮扶机制，重点推进“就业、技能、社保、人才”精准扶贫，确保扶贫对象就业有岗位、创业有资助、技能有提升、参保有安排、医疗有保障。持续提高城乡最低生活保障、特困人员供养、孤儿供养、城乡医疗救助、困难残疾人生活补贴和重度残疾人护理补贴等底线民生保障水平，实现低保标准城乡一体化，完善重特大疾病医疗救助制度。深入开展“携手奔小康”行动，推进大新县、宁明县通过广西壮族自治区“脱贫摘帽”验收，天等县达到国务院“脱贫摘帽”标准。

打响侨乡文化品牌。今年是“公共文化事业建设年”，要深入挖掘、保护与传承侨乡文化，加快创建中国（江门）侨乡文化生态保护实验区，办好2019年中国（江门）侨乡华人嘉年华活动，实施华侨华人名人宣传推广计划、“侨文化寻根”工程，继续推进海丝史迹申遗。擦亮陈白沙、梁启超、冯如等文化名片。发展新乡贤文化，推进文化祠堂建设。实施侨乡文艺精品提升工程，推进文化产业转型发展。加大公共文化场馆建设力度，加快五邑华侨华人博物馆创建国家一级博物馆工作，推进五邑图书馆改扩建项目建设，进一步完善市县镇村四级公共文化设施网络，推进基层综合性文化服务中心建设，全面实现公益文化设施免费开放。加快市体育运动学校扩容提质工程建设，充分利用江门体育中心的先进场馆设施，申办国际五人足球赛，着力打造具有国际影响力的大型赛事重要基地。推动传统武术进校园。加快推进江门市档案中心、全市档案馆达标和全市数字档案馆（室）项目建设。

深入建设“平安江门”。加强社会治安重点整治，严厉打击各类违法犯罪活动，深入开展扫黑除恶专项斗争。全面实施智慧新警务工程，建成市反恐指挥中心，推进“雪亮工程”建设。启动全市困难派出所专项帮扶三年计划。健全“大禁毒”机制，建成市病残吸毒人员收治所。严格执行维稳综治及安全生产党政同责、一岗双责、齐抓共管、失职追责，深化风险排查管控，坚决遏制较大以上事故发生。完善突发事件应急机制，全面提升防灾减灾救灾能力，加强气象综合监测体系建设，提高预报服务能力。深化放心消费创建活动。健全社会矛盾纠纷预防和多元化解机制，推进“全科网格”服务管理体系建设，着力把“鹤城模式”打造成“枫桥经验”的江门样本。全面推进江海区全国农村社区治理实验区建设。

做好上述民生工作，必须加大民生投入，全市各级财政安排民生支出282.36亿元，增长9%。抓好民生实事落实，补齐民生社会事业短板，着力构建共建共治共享社会治理格局。进一步完善国防动员体系，继续做好外事、台港澳、民族、宗教、人防、优抚、打私、统计、信访、地方志、妇女儿童、红十字会等工作。积极推进与四川省甘孜州的对口支援、与黑龙江省七台河市的对口合作。

五、加强政府自身建设

做好新一年政府工作，必须落实全面从严治党要求，树牢“四个意识”，坚定“四个自信”，坚决做到“两个维护”，全面加强政府自身建设，深入推进政府职能转变，提升担当履职水平。

强化政府系统党的建设。严守政治纪律和政治规矩，始终做到“五个必须”，坚决反对“七个有之”，做到“三个决不允许”，全面彻底肃清李嘉、万庆良恶劣影响。在市委的领导下，不断加强市政府党组建设，发挥各级政府党组织在抓好业务工作中的引领作用，严格落实“三重一大”事项集体决策制度。实施“亿元保障计划”，建立市、县、镇三级党建工作经费投入逐年增长机制并且纳入各级财政预算。加强国企基层党组织建设，推进民营企业和外资企业的党建工作，抓好社会组织领域党的组织和工作覆盖。持续加强和改进政府系统意识形态工作。

强化法治政府建设。促进政府依法行政。主动接受人大监督，认真执行市人大及其常委会各项决议决定，支持政协委员参政议政，提高人大代表议案、建议和政协提案办理水平。落实市人大常委会年度立法计划，继续深入推进政府规章和政府规范性文件制定及备案审查工作，落实人大代表票决民生实事项目。支持市中级法院、市检察院工作，完成市县两级行政执法体制改革，严格规范公正文明执法。进一步加强公共法律服务体系建设，优化升级市县镇村四级公共法律服务平台。进一步深化政务公开，全面推进阳光政务建设。

强化高效政府建设。大力倡导“马上就办、办就办好、真抓实干、滴水穿石”的工作作风，主动对接大湾区政务服务规则和标准。加快推进“数字政府”综合改革试点工作，运用大数据提升政府治理能力。全面推进政务服务“马上办、网上办、就近办、一次办”落地，深度融合线下实体服务厅与线上虚拟服务厅，四级政务大厅探索推行“5+X”工作日服务，确保市级、县级政务服务事项网上可办率分别超过85%和70%。开展“不动产登记+互联网+开发企业服务”和不动产登记自助办理模式。提升“12345”政府服务热线水平，组建全市统一的智慧城市管理指挥中心。全面完成市县政府机构改革各项工作任务，深化镇（街）行政管理体制改革。推进承担行政职能事业单位改革和生产经营类事业单位改革。

强化廉洁政府建设。严格落实各级政府、各部门党风廉政建设责任制，落实党内监督条例，支持市监察委员会工作，加强审计和巡察结果运用，持之以恒正风肃纪，保持惩治腐败高压态势。深入查找和纠正“四风”突出问题特别是形式主义、官僚主义的新表现，不断巩固和拓展落实中央八项规定精神的成果。着力解决行政执法、土地出让、审批监管、项目投资、工程招投标等重点领域的违法违规问题，大力整治群众身边的不正之风，构建“亲”“清”新型政商关系，营造风清气正的良好政治生态。

各位代表，习近平总书记视察广东的重要讲话精神，为我们在新时代开创江门政府工作新局面指明了前进方向，让我们更加紧密团结在以习近平同志为核心的党中央周围，在省委、省政府和市委的坚强领导下，凝心聚力、团结奋进，闻鸡起舞、日夜兼程、风雨无阻，全力以赴完成市人民代表大会确定的各项目标任务，加快建设“四个走在全国前列”示范城市，向侨乡人民交出一份满意的答卷，以优异的成绩迎接中华人民共和国成立70周年。
